# Review of Microchip Analytical Methods Coupled with Aptamer-Based Signal Amplification Strategies for High-Sensitivity Bioanalytical Applications

**DOI:** 10.3390/bios15100653

**Published:** 2025-10-01

**Authors:** Xudong Xue, Yanli Hou, Caihua Hu, Yan Zhang

**Affiliations:** 1 Xi’an Innovation College of Yan’an University, Xi’an 710100, China; 2 Faculty of Science, Kunming University of Science and Technology, Kunming 650500, China

**Keywords:** microchip, aptamer, signal amplification strategies, protein, cells, bacteria

## Abstract

Aptamers have many advantages, including facile synthesis and a high affinity and good selectivity toward their targets. Therefore, aptamer-based biosensors have become increasingly popular for the detection of different bioanalytical substances. Microchip-based analytical detection platforms offer significant advantages for the detection of different analytes, including their ease of operation, high throughput, cost-effectiveness, and high sensitivity. Aptamer-based signal amplification techniques have been combined with microchips to sensitively detect bioanalytical substances due to their stable reactions, easy operation, and specificity in biomedical science and environmental fields. This review summarizes representative articles about aptamer signal amplification strategies on microchips for the detection of bioanalytical substances, as well as their advantages and challenges for specific applications. We highlight the accomplishments and shortcomings of aptamer signal amplification strategies on microchips and discuss the direction of development and prospects of aptamer signal amplification strategies on microchips.

## 1. Introduction

Aptamers are highly structured oligonucleotide chains that can be screened by exponential enrichment systems [1]. Since RNA aptamers were first developed in the early 1990s [2,3], many aptamers have been screened, including small molecules [4,5], macromolecules [6,7], proteins [8,9], microorganisms [10,11], and cells [12,13]. Aptamers can fold into various structures, including pseudoknots, stem-loops, hairpins, bulges, and G-quadruplexes. Aptamers bind to their targets with high affinity and specificity via hydrogen bonding, van der Waals forces, and electrostatic interactions. [14]

More importantly, the affinity of an aptamer is similar to that of an antibody directed against a target molecule. However, unlike antibodies, aptamers have a remarkable advantage that is facile synthesis [15,16]. Aptamers are also more stable than antibodies over a wide range of temperatures, pH, and salt concentrations [17]. Functional groups can be tailored to different purposes without changing the intrinsic characteristics of aptamers. Based on these advantages and their biochemical properties, aptamers have been used to develop biosensor platforms for disease diagnosis and biological sensing [18], including aptamer-based microchip strategies for detecting bioanalytical substances due to their simple operation and high sensitivity [19,20].

Microchips have received attention for the bioanalytical detection of substances in food, blood, and cancer. For example, aptamers can be combined with microchips to serve as potential biosensor platforms for environmental monitoring and food safety [21]. Thus, various microchip platforms have been developed, including paper-based microchips [22], microfluidic chips [23], and microarray chips [24]. These microchips have short detection times, require small sample volumes, and are easy to operate and portable. However, high-sensitivity detection is still challenging. Aptamer-conjugated microchips provide a promising strategy for the high-sensitivity detection of the analysis of biologically relevant substances.

**Figure 1:**
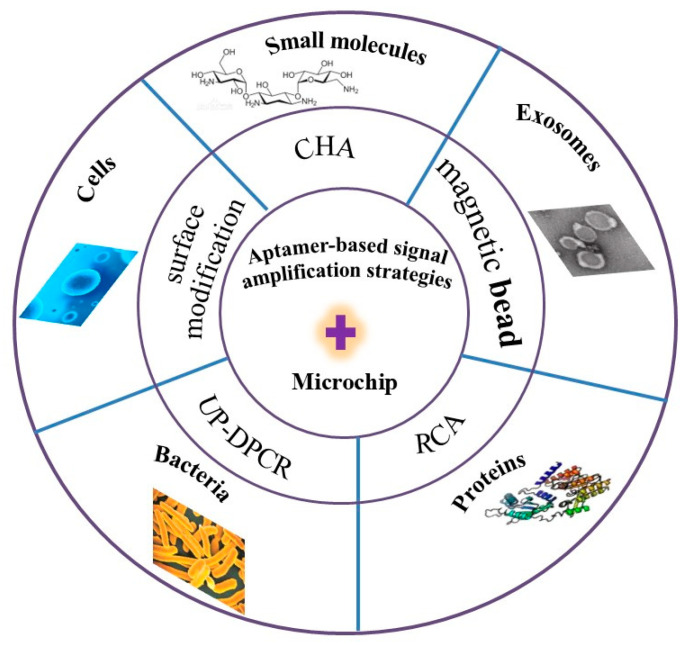
Schematic illustration of the review paper.

Numerous microchip approaches for detecting bioanalytical substances have been reported [19,20,25,26], including those that combine aptamers with microchip methods. Because microchip detection allows the results to be directly observed in the form of spectra, we have focused on aptamer-based strategies for high-sensitivity analysis of bioanalytical substances using microchips. Therefore, this article reviews research progress in the monitoring of small molecules, macromolecules, proteins, microorganisms, and cells, as well as the generation of new sensor signals based on aptamer-based signal amplification on microchips in recent years (Figure 1). The main challenges, future prospects, and development trends in the practical application of bioanalytical substances analysis are also discussed.

## 2. Signal Amplification Strategy Based on Aptamer-Integrated Microchips

### 2.1. Detection of Small Molecules Using Amplification Strategies Based on Aptamer-Integrated Microchip

**Figure 2:**
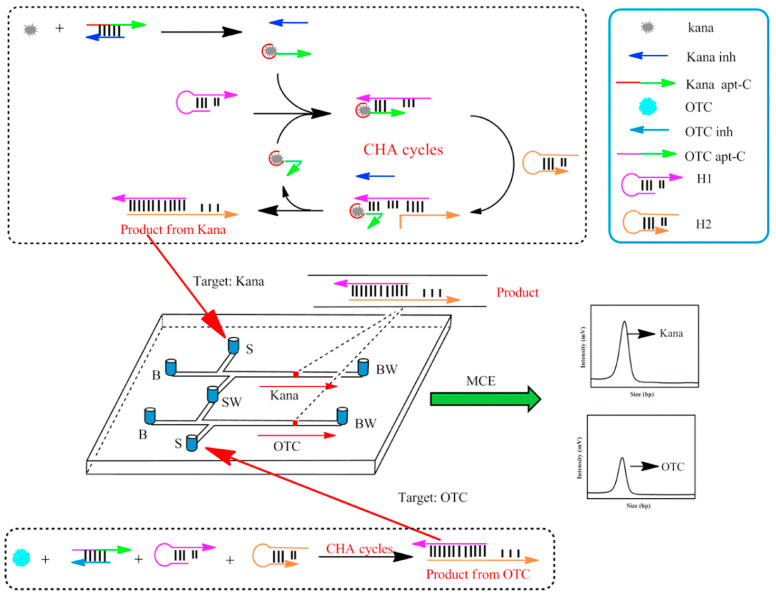
Schematic of the detection of kanamycin (Kana) and oxytetracycline (OTC) based on catalyzed hairpin assembly (CHA) coupled with MCE [4].

The overuse of antibiotics has side effects on human health. The reason is that rampant use of antibiotics can lead to some serious drug-resistant diseases [27]. Until now, antibiotics have mainly been detected using methods such as liquid chromatography [28], electrochemical sensors [29], and fluorescence detection [30]. However, the high-sensitivity detection of antibiotics poses an issue due to their low concentration and matrix interference in samples. Thus, some of the above strategies may not be suitable for detecting antibiotics in complex matrixes or fast and high-throughput analysis in food safety screening. Sensitive and selective aptamer probes have been used as sensors for the specific recognition and detection of small molecules in complex matrixes. Antibiotics have been detected by multiple types of aptasensors such as fluorescent [31], electrochemical [32], and colorimetric methods [33]. With the development of microchip technologies, antibiotics have been detected by many researchers based on aptamer-conjugated microchips. Zhou et al. reported a rapid, sensitive, and high-throughput platform for the detection of chloramphenicol (CAP) by combining aptamer probes with microchip electrophoresis. First, the aptamer probe (Apt) was selected by capturing CAP, which was employed as a model. The partial complementary oligonucleotide of CAP’s aptamer (C-DNA) was chosen for the reaction system. When CAP was present, its aptamer was bound to it, which prevented the aptamer from hybridizing with C-DNA to form dsDNA. Thus, when the target to its CAP was present or not, the amount of ssDNA and dsDNA was separated and detected by microchip electrophoresis (MCE) based on the different base pairs for the Apt-CAP and dsDNA. The different fluorescence signals were expressed as Apt-CAP and dsDNA, respectively, and targets were detected based on the ratio of the aptamer-CAP and dsDNA signals [34]. Wang et al. developed a novel aptasensor based on an MCE platform for the simultaneous detection of several antibiotics using the scheme illustrated in Figure 2. In the presence of Kanamycin (Kana) and oxytetracycline (OTC), the targets produced strong signals by the catalyzed hairpin assembly (CHA) method, which achieved a 300-fold amplified signal compared with the non-amplified system. Under optimal conditions, the MCE platform obtained kanamycin and oxytetracycline limits of detection of 0.7 pg mL^−1^ and 0.9 pg mL^−1^ [4]. Zhou et al. developed a novel MCE array-based aptasensor for the multiplex detection of antibiotics that used multi-capture DNA functionalized magnetic beads and polymerase chain reaction (PCR) for signal amplification. Under optimal conditions, the limits of detection for kanamycin (KANA) and chloramphenicol (CAP) were 0.0025 nM and 0.006 nM, respectively [35]. Zhang et al. developed a multiplexed aptasensor for the one-step and simultaneous detection of several antibiotics based on a universal double-T-type microchip. The advantage of this strategy was that the stir bar facilitated phase separation and matrix interference in food samples. The results showed excellent selectivity and sensitivity, with a limit of detection of 0.52 pg mL^−1^ chloramphenicol and 0.41 pg mL^−1^ kanamycin [36]. Zhang et al. described an enzyme-free aptamer-based assay for the detection of antibiotic kanamycin that used a stir bar carrying a gold-labeled aptamer coupled with MCE platform and hybridization chain reaction (HCR) signal amplification. In the presence of kanamycin, the complementary chains (cDNA) were released when the aptamer on the stirring bar unwound the duplex DNA. The released cDNA triggered the HCR in the presence of H1 and H2 DNA hairpin. The amounts of H1 and H2 decreased, and kanamycin was quantified based on a decrease in the H1/H2 signal ratio. Kanamycin was detected in the range of 1 pg mL^−1^ to 10 ng mL^−1^ with a limit of detection of 0.29 pg mL^−1^ [37]. Chen et al. developed a ratiometric and homogeneous assay for kanamycin detection using MCE. The design of a novel R-shaped DNA probe (R-DNA) containing one single-stranded DNA (S-DNA) and one hairpin DNA (H-DNA). When the R-DNA probe was incubated with KANA, the S-DNA-KANA complex was formed, and H-DNA was released. The S-DNA-KANA complex was digested by Exonuclease I (Exo-I), and the released KANA was once again able to trigger target recycling for signal amplification. The produced H-DNA and remaining R-DNA could be easily separated and detected by MCE, resulting in a limit of detection of 150 fg mL^−1^ for kanamycin [38]. Zhou et al. developed a simple double-T-type MCE platform for the detection of ampicillin (AMPI), adenosine triphosphate (ATP), and estradiol (E2) using an aptamer probe. The probe used isothermal polymerase-catalyzed target recycling (IPCTR) for signal amplification. Under optimal conditions, this approach exhibited high sensitivity to three targets in urine with limits of detection of 0.05 nM (AMPI), 1 nM (ATP), and 0.1 nM (E2) [39].

**Figure 3:**
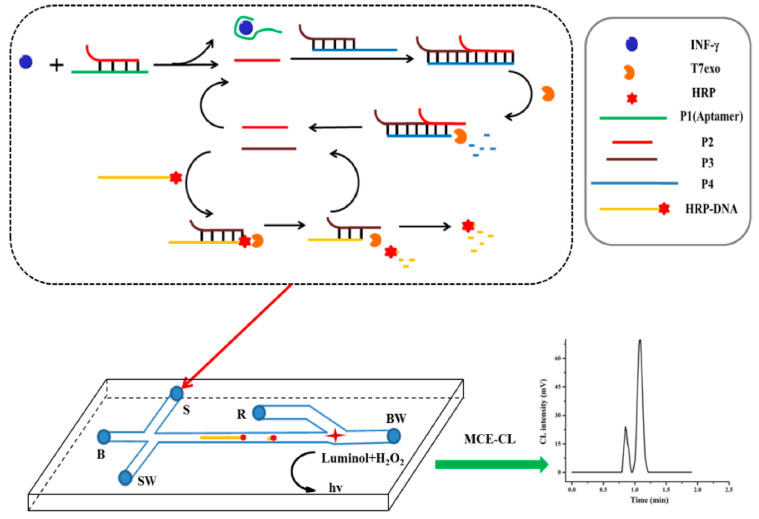
The ultrasensitive detection of gamma interferon (IFN-γ) based on an MCE platform coupled with target triggering cascade CL signal amplification [40].

To enable the early diagnosis of diseases using the MCE platform, an ultrasensitive microchip electrophoresis chemiluminescence (MCE-CL) assay platform was developed to detect trace biomolecules. Yang et al. developed an MCE-CL assay platform utilizing nucleic acid hybridization and exonuclease cutting technology for the ultrasensitive detection of gamma interferon (IFN-γ) with a limit of detection of 1.6 fM (Figure 3) [40].

The detection of trace tumor markers (TMs) is important for the early diagnosis of cancer, but it is challenging to fabricate assays for simultaneously detecting trace levels of TMs. Xie et al. developed a novel MCE method and antibody–aptamer-based hybrid detection strategy for the simultaneous determination of carcinoembryonic antigen (CEA), prostate-specific antigen (PSA), and carbohydrate antigen 125 (CA125) in human serum. This approach was based on the generation of magnetic aptamer-capture probes coupled with anti-TMs labeled encoded signal tags for signal amplification using a nicking enzyme strategy. The resulting limits of detections were 0.15, 0.1, and 0.12 pg mL^−1^ for CEA, PSA, and CA125, respectively [41].

### 2.2. Detection of Exosomes Using Amplification Strategies Based on Aptamer-Integrated Microchip

**Figure 4:**
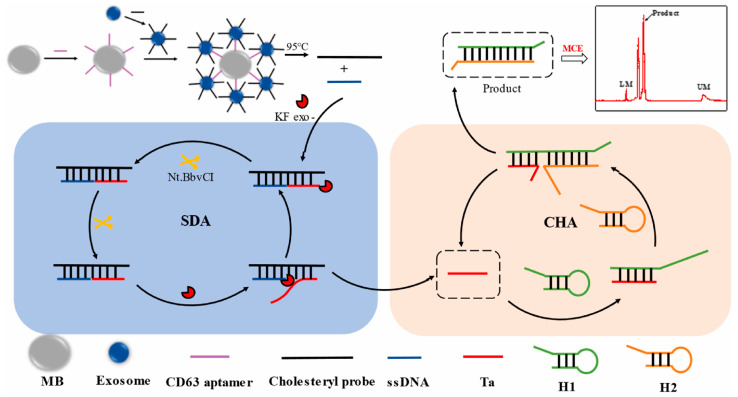
Schematic of the detection of exosomes based on triple amplification strategies with MCE [55].

Exosomes are nano-vesicles in the presence of blood, urine, and saliva. They were first reported in the early 1980s, and have received great attention between exosomes and tumors [42,43]. Many bioactive substances, such as proteins and nucleic acids exist in exosomes, and the properties of these bioactive substances are consistent with their parent cells [44]. Exosomes can also regulate immune functions or directly act on cells to promote tumor angiogenesis and metastasis. More exosomes are released in tumor cells compared with normal cells [45]. Exosomes have significant effects on adaptive immunity, embryogenesis, inflammatory processes, and tumorigenesis [46] and are therefore used as biomarkers for early cancer diagnosis. A series of analytical techniques, such as flow cytometry [47], protein blotting (Western blotting, WB) [48], and ELISA [49], have been developed to extract, characterize, and quantify exosomes. In addition, the amount and size distribution of exosomes have been determined by mass spectrometry (MS) [50], surface plasmon resonance (SPR) [51], surface-enhanced Raman scattering (SERS) [52], and nuclear magnetic resonance (NMR) spectroscopy [53]. MCE has also been used to analyze exosomes due to its short detection time, low sample consumption, convenient operation, and high separation efficiency. The combination of microfluidic chips and aptamers is also a popular strategy. Chen et al. first developed a quantitative MCE strategy combining cholesterol probe (Chol-probe) with strand displacement amplification (SDA)-catalytic hairpin assembly (CHA) to detect human breast cancer cell (MCF-7) exosomes. The schematic of this exosome-based detection method is shown in Figure 4. In the first step of the amplification strategy, the CD63 aptamer was immobilized on the avidin magnetic beads and used to capture exosomes based on CD63 aptamer. Then, a high-affinity Cho-probe was inserted into the exosome membrane. After magnetic separation, the Chol-probe could initiate the SDA reaction to produce numerous short-stranded DNA (Ta) under the action of polymerase and a nicking enzyme. The obtained Ta triggered the CHA, thereby achieving SDA-CHA amplification and obtaining a limit of detection of 26 particle μL^−1^ [54]. Zhao et al. proposed an automated centrifugal microfluidic disc system that was combined with functionalized membranes (Exo-CMDS) to isolate and enrich exosomes. This system utilized a novel aptamer magnetic bead bioconjugate with an HRP-probe attached. The purified exosomes were captured by the aptamers, and a fluorescent signal was produced by HRP catalyzing the reaction between hydrogen peroxide and Amplex Red. The spectral intensity was related to the level of the specific exosome protein, and the exosomal concentration was determined to be 5.1 × 10^9^ particles mL^−1^ [55].

### 2.3. Detection of Proteins Using Amplification Strategies Based on Aptamer-Integrated Microchip

**Figure 5:**
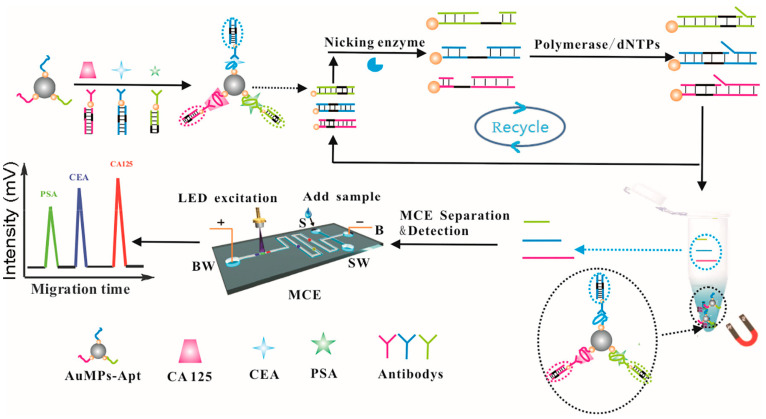
Schematic of the detection of PSA, CEA, and CA125 based on antibody–aptamer coupled with a microfluidic chip [41].

Trace levels of proteins play an important role in the biological functions of cells [56,57], making it important to rapidly and sensitively detect proteins [58]. Traditional solid-phase protein detection strategies include high-performance liquid chromatography (HPLC) [59], enzyme-linked immunity assays [60], and magnetic-separation assays [61]. However, these methods detect target proteins by relying on antibody specificity, and antibodies are expensive, unstable, and often difficult to source. In addition, non-specific adsorption and cross-reactions also influence traditional solid-phase protein detection strategies. An aptamer-based signal amplification coupled with a microchip provides an attractive strategy for the detection of proteins. Wang et al. reported a chemiluminescence assay with dual-signal amplification for the ultrasensitive detection of thrombin on microchips based on multiDNAzymes-functionalized gold nanoparticles (AuNPs) using in situ rolling circle amplification (RCA). The detection sensitivity was greatly improved using this signal amplification strategy. The chemiluminescence assay of thrombin achieved a linear range of 1–25 pM, with a limit of detection of 0.55 pM [62]. Lin et al. described a simple and rapid strategy for detecting multiplex proteins (PDGF-BB and VEGF_165_) based on tunable aptamers by microchip electrophoresis. The mechanism involved modulating the electrophoretic mobility of proteins by changing the aptamer length. Lin et al. reported a method in which the concentration of PDGF-BB and VEGF_165_ was detected in the ranges of 5.15 nM to 2.03 nM, and 3.14 to 2.53 nM, respectively [63]. Pan et al. developed an aptamer-based MCE strategy for assaying carcinoembryonic antigen (CEA) in human serum for cancer diagnosis. Double-stranded DNA, formed by an aptamer of the target and a complementary DNA of the aptamer, was adsorbed on the magnetic beads (MBs). When the target bound to the aptamer in the MB–dsDNA conjugate, the complementary DNA was released from the MB–dsDNA conjugate. The fluorescein amidite (FAM)-labeled DNA was hybridized with the released complementary DNA to form a DNA duplex that then triggered the selective cleavage of FAM-labeled DNA by nicking endonuclease Nb.BbvCI. This was accompanied by obtaining a FAM-labeled DNA segment, and then another FAM-labeled DNA was hybridized with the released complementary DNA. The obtained result was that FAM-labeled DNA was continuously cleaved, thus generating large numbers of FAM-labeled DNA segments that could then be separated and detected by MCE [64]. Bhardwaj et al. developed a method for the inline monitoring of Ranibizumab in bioreactors by coupling an aptamer with a microfluidic chip. The selected aptamers originated from 10 rounds of the SELEX process, which exhibited high affinity towards Ranibizumab. The detection efficiency of this method was better than HPLC [8]. The detection of trace levels of tumor markers (TMs) can provide more accurate information and higher efficiency for the early diagnosis of cancer, but the simultaneous detection of trace levels of TMs is still challenging. Against this background, MCE analysis methods with a high sensitivity and high throughput for the simultaneous detection of different TMs have emerged. Xie et al. developed a novel MCE- and antibody–aptamer-based hybrid detection strategy for the simultaneous determination of carcinoembryonic antigen (CEA), prostate-specific antigen (PSA), and carbohydrate antigen 125 (CA125) in human serum, whose detection mechanism is shown in Figure 5. The aptamers of TMs were used as capture probes and were co-immobilized on surface of Fe_3_O_4_@AuNPs. The encoded signal tags were composed of the antibodies of TMs labeled with different double-stranded DNA (dsDNA) as nicked fragment-induced strands. Then, the encoded signal tags, capture probes, and TMs formed sandwich complexes under simultaneous incubation. After magnetic separation, the complex was formed by adding a nicking enzyme. Many single-stranded DNA (ssDNA) products, whose different lengths corresponded to different targets, were produced from the dsDNA on the complex initiating nicking enzyme cleavage reaction. The corresponding target of ssDNA products were separated and detected by MCE. This strategy exhibited distinct advantages with limits of detection of 0.15, 0.1, and 0.12 pg mL^−1^ for CEA, PSA, and CA125, respectively [41].

**Figure 6:**
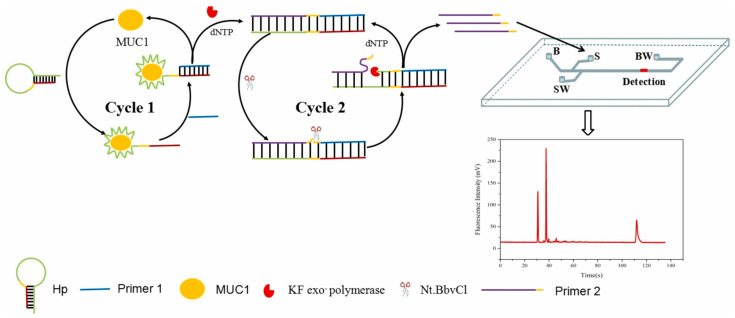
Schematic of the detection of MUC1 based on TRA and SDA combined with MCE [65].

Mucin 1 (MUC1) is abnormally expressed in cancers, allowing it to be used as a tumor marker for the early diagnosis of cancer. Therefore, establishing an ultrasensitive and rapid method of MUC1 detection is meaningful. Geng et al. reported a highly sensitive MUC1 assay in which MCE was coupled with target recycling amplification (TRA) and strand displacement amplification (SDA) (Figure 6). In this strategy, a hairpin probe (HP) was designed with the aptamer of MUC1, the recognition site for Nt.BbvCI, and the sequence complementary to primer 1 (P1). When MUC1 was present, the exposure of the designed hairpin probe (HP) was triggered by MUC1 because MUC1 was bound to the aptamer. Then, a large number of ssDNA were produced and detected by MCE. The MUC1 concentration was determined by correlating the MUC1 concentration and ssDNA concentration. The ssDNA concentration was used to indicate the concentration of MUC1 according to the correlation between MUC1 concentration and ssDNA concentration. The results showed a good linear relationship with MUC1 concentration in the range of 1.0 pg/mL–1.0 × 10^3^ pg/mL with a low limit of detection of 0.23 pg/mL [65]. Yang et al. developed an ultrasensitive MCE-CL assay platform for the detection of trace biomolecules, thus realizing the early diagnosis of diseases using the MCE platform. In this work, the aptamer was used to bind a target molecule to trigger a cascade signal amplification reaction. The target probe was the aptamer, and the signal probe was horseradish peroxide-labeled DNA (HRP-DNA). The method utilized nucleic acid hybridization and exonuclease cutting to realize the ultrasensitive detection of biomolecules on the MCE-CL assay platform. Gamma interferon (IFN-γ) was detected with a limit of detection of 1.6 fM, thus enabling its quantification in human plasma samples [40]. Wang et al. developed a chemiluminescence assay with a dual signal amplification strategy for the detection of thrombin on a microchip. This strategy was based on multiDNAzyme-functionalized gold nanoparticles (AuNPs) using in situ rolling circle amplification (RCA), which achieved a linear range of 1–25 pM and a limit of detection of 0.55 pM [62].

### 2.4. Detection of Bacteria Using Amplification Strategies Based on Aptamer-Integrated Microchip

Pathogenic bacteria must be detected to ensure food safety and public health because they pose threats to public health around the world [66]. The ability to detect pathogen bacteria is of great significance for preventing the spread of illnesses. The most frequently used method is the standard plate colony counting method, which is time-consuming and requires several days to obtain results [67]. Therefore, fast and highly sensitive strategies for the detection of pathogenic bacteria are urgently needed. Various detection methods already exist, including ELISA [68], real-time quantitative polymerase chain reaction (qPCR) [69], chemiluminescence (CL) techniques [70], surface-enhanced Raman spectroscopy (SERS) [71], and electrochemical biosensors [72]. Due to the respective advantages of each of these methods (such as miniaturized, low-cost devices with high sensitivity), a powerful analytical technique for microchips that combines their high throughput, separation efficiency, cost-effectiveness, low analyte consumption, high sensitivity, and automated analysis has become popular for the detection of pathogenic bacteria. Zhang et al. reported a simple and sensitive strategy to detect *S. Typhimurium* by MCE based on the specific reaction between the bacterium and corresponding aptamers. The method was based on the different charge-to-mass ratios of the bacteria-aptamer complex and free aptamers. Under the optimal conditions, the limit of detection of 3.37 × 10^2^ CFU mL^−1^ for *S. Typhimurium* was achieved [10]. Zhang et al. detected *E. coli* by using this strategy based on bacteria-specific aptamers [73].

**Figure 7:**
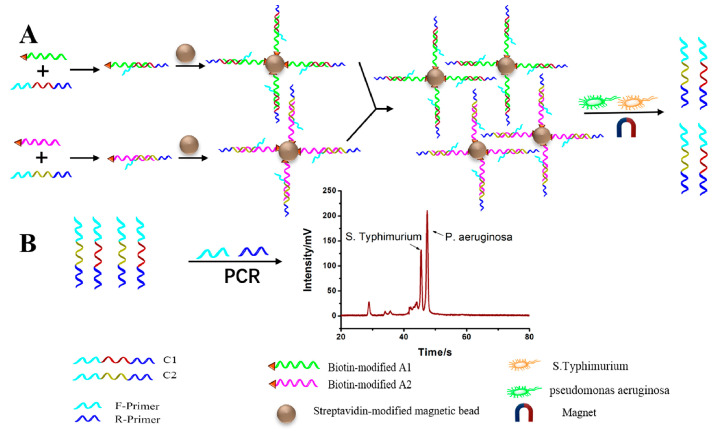
Detection of *S. Typhimurium* and *P. aeruginosa*. (**A**) C1 and C2 were released by Aptamers recognize the bacterial cells. (**B**) Detection of the released C1 and C2 based on UP-DPCR coupled with MCE-LIF [75].

To improve the sensitivity of bacteria detection, aptamer-induced catalyzed hairpin assembly (CHA) was developed using MCE. CHA is a nucleic acid-based cycle amplification method that generates numerous DNA duplexes, allowing the CHA-based MCE to detect pathogenic bacteria. Luo et al. first developed such a strategy to detect *E. coli* O157:H7, in which CHA was combined with MCE to obtain a cost-effective and ultrasensitive method with a limit of detection as low as 75 CFU mL^−1^. The quantification of *E. coli* O157:H7 in defatted milk was achieved with a satisfactory recovery rate [74]. Luo et al. reported a new method in which an aptamer-based probe and a novel universal primer-duplex polymerase chain reaction (UP-DPCR) were combined with the MCE strategy (Figure 7). This strategy simultaneously detected *S. typhimurium* and *Pseudomonas aeruginosa* (*P. aeruginosa*), giving LODs of 15 CFU mL^−1^ and 5 CFU mL^−1^, respectively [75]. Jiang et al. designed a new dual-rolling circle amplification (RCA) approach for whole-cell sensitive detection by using microfluidic devices in which long tandem repeating aptamers captured the target *E. coli* O157:H7. This was used to modify microfluidic channel surfaces with three times as many target bacteria than microchannels modified with mono-aptamers against the target bacteria [76]. Li et al. applied rolling circle amplification (RCA) to modify microfluidic channels for the sensitive detection of *E. coli* O157:H7. This strategy was based on modifying the inner surfaces of microfluidic channels with cRCA products using specific aptamers for *E. coli* O157:H7 [77].

A novel dendrimer-aptamer-coated microfluidic device was employed to detect *E. coli* O157:H7. A poly(amidoamine) dendrimer was immobilized on PDMS microchannels and then an *E. coli* O157:H7-capturing aptamer was immobilized on the poly(amidoamine) dendrimer. In the presence of captured *E. coli* O157:H7, the rolling circle amplification (RCA) reaction was triggered, which resulted in a 50-fold increase in fluorescence intensity than that without RCA. The limit of detection of this system decreased to 10^2^ cells mL^−1^ with excellent specificity [78]. Li et al. developed a fast and sensitive detection method that introduced a visual microfluidic aptasensor (EA-Sensor) for the rapid detection of *E. coli* O157:H7. This method could be used without instrumentation. This method was based on aptamer sensing, hybridization chain reaction (HCR)-amplification, and a distance-based visualized readout to detect the concentration of pathogens. The system was evaluated by gel-electrophoresis assay. The aptamer could recognize *E. coli* O157:H7, and the signal was achieved by about 100 folds based on HCR. Under the optimal conditions, the limit of detection of 2.5 × 10^2^ CFU mL^−1^ for *E. coli* O157:H7 was achieved [79].

### 2.5. Detection of Cells Using Amplification Strategies Based on Aptamer-Integrated Microchip

**Figure 8:**
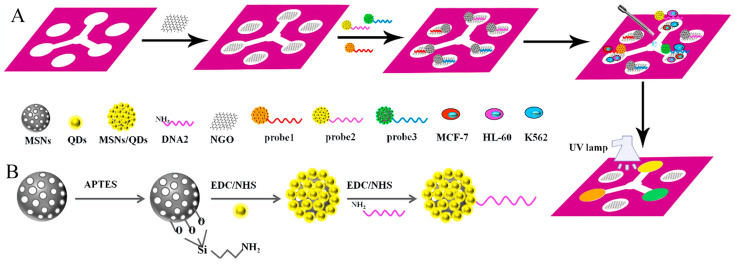
Schematic of the detection of MCF-7 cells based on microfluidic paper-based analytical devices (μ-PADs), including the fabrication procedure (**A**) and entire assembly process of the probe (**B**) [80].

**Figure 9:**
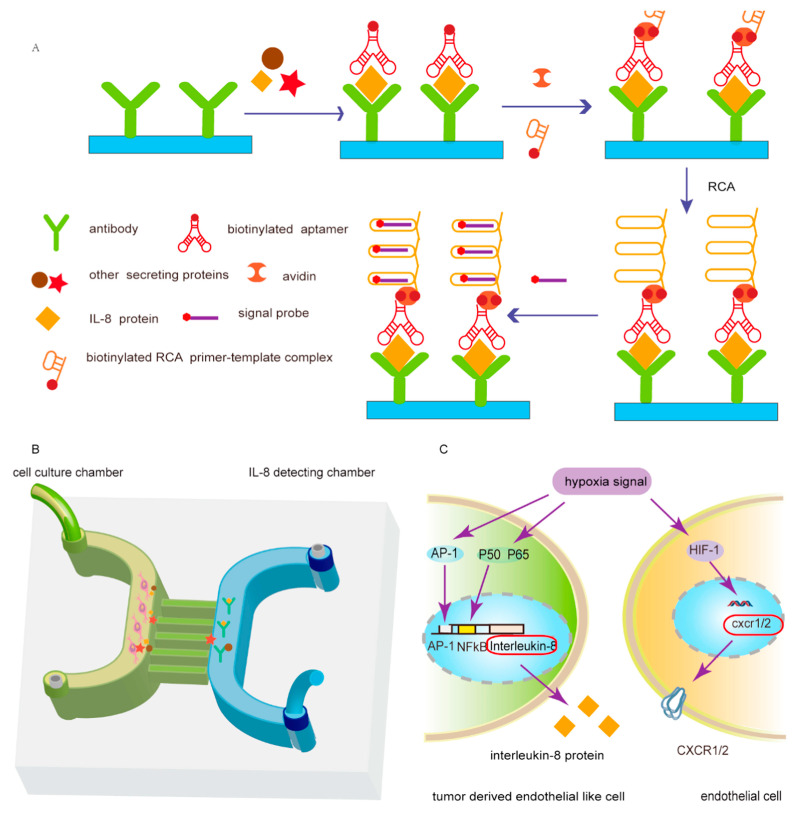
Schematic of the detection of IL-8 based on RCA and fluorescence probe under hypoxic conditions on the microchip [82]. (**A**) The proposed schematic of the detection method of IL-8. (**B**) Dual-function microfluidic chip. (**C**) Hypoxic signal pathway in tumor-derived endothelial cells (TDEC) and human umbilical vein endothelial cells (HUVEC).

Rapid, facile, portable, disposable, and low-cost fluorescence methods for the visual detection of cells using aptamers coupled with microchips are becoming increasingly popular. Particularly for the detection of circulating tumor cells (CTCs), microchips have received attention and faced great challenges. Liang et al. designed a cancer cell-sensing platform for the multiplexed monitoring of cancer cells using graphene oxide (GO)-based aptameric nanosensor in microfluidic paper-based analytical devices (Figure 8). This strategy combined the high specificity and affinity of aptamers with the exceptional quenching capability of GO to recognize element. The selected aptamers were labeled on quantum dot-coated mesoporous silica nanoparticles, wherein the fluorescence was quenched by GO. The fluorescence recovered in the presence of the target cells, and the color changes could be directly observed with the naked eye. This strategy exhibited the limits of detection as low as 62 cells mL^−1^ for MCF-7 cells, 65 cells mL^−1^ for K562 cells, and 70 cells mL^−1^ for HL-60 cells [80]. Sheng et al. developed a platform composed of multivalent DNA aptamer nanospheres combined with microfluidic devices to efficiently capture CTCs. Avidin was first coated on a microfluidic device surface and then the biotinylated aptamer-conjugated AuNPs bound to the avidin based on biotin-avidin interactions. When the target cancer cells were in the channel, aptamers captured them. Due to the binding affinity of AuNP-aptamer conjugates, this strategy obtained a 39-times higher binding affinity compared with the aptamer alone [81]. Zhang et al. reported a dual-function microfluidic biosensor for cell culturing and online IL-8 detection. This method improved the detection sensitivity through the isothermal amplification of nucleic acid aptamers (Figure 9). A microfluidic chip was formed by connecting two high microchannels and several narrow microchannels. One channel was used to culture cells and another was prepared for IL-8 detection based on immobilizing captured antibodies. A sandwich-type immunoassay was designed by antibodies, IL-8, and aptamers. Signal amplification was attributed to the immunoassay of IL-8 based on the biotin-streptavidin linkage and RCA method. The limit of detection was as low as 0.84 pmol L^−1^ for IL-8 [82]. Zhang et al. developed a novel strategy for high-performance CTC capture and detection by combining biomimetic magnetosomes with a nickel-patterned microfluidic device. Leukocyte membrane fragments were coated onto Fe_3_O_4_ magnetic nanoclusters to form biomimetic nanoparticles. SYL3C aptamers were modified on the surface of biomimetic nanoparticles, and the formed complex was magnetically loaded into the microfluidic channel. More than 90% of rare tumor cells in blood could be captured and detected by using this system within 20 min without a leukocyte background [83]. Nguyen et al. captured A549 human lung CTCs using a method that was based on fabricating a microfluidic chip with the aptamer-conjugated self-assembled monolayer (SAM) of gold nanoparticles (AuNPs) in the channel [84]. Nguyen et al. developed a simple microfluidic platform composed of a combination of DNA aptamers and impedance measurements to detect the A549 human lung carcinoma cell line [85]. Khaksari et al. established a novel microfluidic electrochemical aptasensor for the detection of A549 human adenocarcinoma cells. The IDA aptamer as a recognition element was first used in a microfluidic aptasensor. This work was based on the conformational change of IDA aptamer structure with an affinity towards A549 cells. The combination of a screen-printed gold electrode functionalized with SH-IDA aptamers and a microchip was performed, and the microfluidic biosensor showed a limit of detection as low as 14 cells mL^−1^ for A549 cells [86].

## 3. Conclusions, Future Prospects, and Challenges

In the past few decades, various aptamer-based sensors for the detection of different analytes have received increasing attention [87,88,89,90]. This article reviewed research progress in the monitoring of small molecules, macromolecules, proteins, microorganisms, and cells, as well as the generation of new sensor signals based on aptamer-based signal amplification on microchips in recent years (Table 1). Aptamer-based signal amplification techniques on microchips have been proposed to sensitively detect substances due to their stable reactions, easy operation, and specificity. The rapid, on-site detection of substances, particularly pathogenic bacteria, has been enabled, but there are still some challenges. For example, the activity of nanomaterials will become inefficient due to denaturation, degradation, or aggregation over time. This will greatly affect the detection performance of microchip sensors. Food samples are complex composite matrix systems, and different nutrients can interfere with the detection of substrates such as pathogenic bacteria, antibiotics, and small molecules. Non-specific binding between the aptamer and sample components may lead to false positives. Aptamer-based microchip sensors also require the samples to be pre-treated before detection in most cases. Magnetic beads or magnetic nanoparticles are often used in these sensors, and although immunomagnetic separation can be used to isolate and concentrate pathogenic bacteria from food matrices, it prolongs the detection time. Integrating magnetic separators with portable devices is also difficult. Microchip sensors based on aptamers perform well under laboratory conditions, but under harsh conditions, aptamers are easily destroyed and degraded. Aptamers may also non-specifically bind to other components in food, resulting in inaccurate results.

Some challenges need to be overcome in the future. For the detection of bacteria and proteins, increasing the sensitivity of aptamer-based microchip remains the primary goal. Therefore, the development of new materials, especially nanomaterials, that combine various signal amplification strategies will make it possible to simultaneously detect molecules, bacteria, and proteins. Specificity is another key issue that needs to be considered for the detection of substances using aptamer-based microchip biosensors. Therefore, researchers should focus on specificity. One of the key factors in constructing microchip biosensors based on aptamers is the specific binding between the target and aptamer. To use aptamers more effectively, it is necessary to study the binding mechanisms, binding sites, and folding structures (such as G-quadruplex structures, convex ring structures, and hairpin structures) of newly screened aptamers with targets. In the face of pathogenic bacteria, aptamer-based microchip biosensors can only detect the overall number of pathogenic bacteria in samples and cannot distinguish between live and dead bacteria. Thus, their detection results may produce false positives. Therefore, developing new strategies to enable biosensors to distinguish between live and dead bacteria is another key focus of research. To detect multiple small molecules, bacteria, or proteins, experiments require different test tubes, which consume more reagents, samples, and time. If multiple substances are detected simultaneously in a test tube, they may interfere with each other and reduce the accuracy of detection. How to efficiently, quickly, and accurately detect multiple targets simultaneously should be a focus of future research. In short, the detection of multiple substances is moving towards automation and miniaturization. In the future, microchip biosensors based on aptamers may simultaneously achieve specific and automated detection of thousands of different samples.

The detection of the key biomarkers is necessary for ultra-trace bioanalysis to realize early-stage diagnosis and therapy. In actual samples, ensuring a sufficient sensitivity and specificity of aptamer-based signal amplification on microchips is still challenging. In addition, the corresponding aptamers for biomarkers with high specificity and affinity have not yet been screened. Due to the complexity of the fabrication process of traditional microchips, there is a need to simplify the production process of microchips. The high-sensitivity detection of these techniques for aptamer-based signal amplification can already be achieved, but the process of binding and signal amplification between the aptamer and a target molecule requires a long amount of time. Therefore, there is a need to improve the signal amplification reaction efficiency and reduce the reaction time. With aptamer-based microchips, background interference needs to be removed to increase the sensitivity. Despite the remaining challenges, aptamer-based microchips show great promise for biomedical and environmental monitoring applications.

Finally, the higher requirements for signal enhancement are put forward by the above prospective viewpoints. The development of signal amplification approaches needs to be explored by researchers. Based on current research, the detection performance has been improved through the signal amplification strategy. However, improving sensitivity remains a primary pursuit in developing signal amplification strategy. The actual detection requirements demand more exploration. The establishment of standard for effective and low-cost aptamer-integrated microchips is driven by universal popularization and application demands. Until now, accuracy and stability in practice detection for the aptamer-integrated microchips remain problems that need to be overcome. There is no doubt that signal amplification strategy based on aptamer-integrated microchips will make distinguished contributions in practical application research.

## Figures and Tables

**Table 1:** Summary of bioanalytical applications based on aptamer signal amplification strategy for microchips.

Applications	Target	Signal Amplification	Linear Range	LOD	Reference
**small molecules**	chloramphenicol	combining aptamer and MCE	0.008–1.0 ng/mL	0.003 ng mL^−1^	[34]
	Kana and OTC	CHA	0.001–10.0 ng/mL	0.7 pg mL^−1^ and 0.9 pg mL^−1^	[4]
	KANA and CAP	magnetic beads and PCR	0.0025–10 nM and 0.006–10 nM	1.2 pg mL^−1^ (0.0025 nM) and 1.9 pg mL^−1^ (0.006 nM)	[35]
	CAP and Kana	a stir-bar assisted multi-arm junctions recycling	0.001 to 40 ng mL^−1^	0.52 pg mL^−1^ and 0.41 pg mL^−1^	[36]
	Kana	HCR	1.0 to 10.0 ng mL^−1^	0.29 pg mL^−1^	[37]
	Kana	exonuclease assisted signal amplification	0.5 to 10.0 ng mL^−1^	150 fg mL^−1^	[38]
	AMPI, ATP, E2	isothermal polymerase-catalyzed target recycling	1–100 nM, 0.05–10 nM, and 0.1–50 nM	0.017 pg mL^−1^ (0.05 nM), 5.07 pg mL^−1^ (1 nM), and 0.027 pg mL^−1^ (0.1 nM)	[39]
	PSA, CEA, and CA125	polymerization nicking reactions	1 pg mL^−1^–1.0 ng mL^−1^	0.1 pg mL^−1^, 0.15 pg mL^−1^ and 0.12 pg mL^−1^	[41]
**exosomes**	human breast cancer cell	SDA and CHA	44–8.75 × 10^5^ particle μL^−1^	26 particle μL^−1^	[54]
	PD-L1	magnetic bead	---	5.1 × 10^9^ particles mL^−1^	[55]
**proteins**	thrombin	RCA	1–25 pM	0.55 pM	[62]
	PDGF-BB and VEGF_165_	micellar enrichment	5.15–2.03 nM, and 3.14–2.53 nM	5.00–150.0 nM	[63]
	CEA	magnetic bead assisted target-induced strand circle	0.13–8.0 ng mL^−1^	0.068 ng mL^−1^	[64]
	MUC1	TRA and SDA	1.0–1.0 × 10^3^ pg mL^−1^	0.23 pg mL^−1^	[65]
**bacteria**	*S. Typhimurium*	micellar enrichment	1.2 × 10^3^–7.5 × 10^5^ CFU mL^−1^	3.37 × 10^2^ CFU mL^−1^	[10]
	*E. coli* O157:H7	CHA	2 × 10^2^–2 × 10^5^ CFU mL^−1^	75 CFU mL^−1^	[74]
	*S. typhimurium* and *P. aeruginosa*	UP-DPCR	5 × 10^1^–5 × 10^6^ CFU mL^−1^ and 1 × 10^1^–5 × 10^4^ CFU mL^−1^	15 CFU mL^−1^ and 5 CFU mL^−1^	[75]
	*E. coli* O157:H7	RCA	10^2^ to 10^5^ cells mL^−1^	120 cells mL^−1^	[76]
	*E. coli* O157:H7	cRCA	10^2^ to 10^5^ cells mL^−1^	5 cells mL^−1^	[77]
	*E. coli* O157:H7	RCA	10^2^ to 10^5^ cells mL^−1^	10^2^ cells mL^−1^	[78]
	*E. coli* O157:H7	HCR	500–5 × 10^7^ CFU mL^−1^	250 CFU mL^−1^	[79]
**cells**	MCF-7 cells, K562 cells, and HL-60 cells	graphene oxide	180–8 × 10^7^, 210–7 × 10^7^ and 200–7 × 10^7^ cells mL^−1^	62 cells mL^−1^, 65 cells mL^−1^, and 70 cells mL^−1^	[80]
	CTCs	multivalent DNA nanospheres	100–10^5^ cells mL^−1^	100 cells mL^−1^	[81]
	IL-8	RCA	7.5–120 pg mL^−1^	0.84 pmol L^−1^	[82]
	circulating tumor cells	Fe_3_O_4_ magnetic nanoclusters	5–10^3^ cells mL^−1^	5 cells mL^−1^	[83]
	A549 cells and HeLa cells	gold nanoparticles	10^5^–10^6^ cells mL^−1^	5 × 10^5^ cells mL^−1^ and 10^6^ cells mL^−1^	[84]
	A549 cells	a process of self-assembled monolayers	1 × 10^5^–5 × 10^5^ cells mL^−1^	1.5 × 10^4^ cells mL^−1^	[85]
	A549 cells	surface modification	50–5 × 10^5^ cells mL^−1^	14 cells mL^−1^	[86]

## Data Availability

No new data were created or analyzed in this study.
